# *MLPAinter for MLPA interpretation: *an integrated approach for the analysis, visualisation and data management of Multiplex Ligation-dependent Probe Amplification

**DOI:** 10.1186/1471-2105-11-67

**Published:** 2010-01-29

**Authors:** Ronald van Eijk, Paul HC Eilers, Remco Natté, Anne-Marie Cleton-Jansen, Hans Morreau, Tom van Wezel, Jan Oosting

**Affiliations:** 1 Department of Pathology, Leiden University Medical Center, Leiden, The Netherlands; 2 Department of Biostatistics, Erasmus University Medical Center, Rotterdam, The Netherlands

## Abstract

**Background:**

Multiplex Ligation-Dependent Probe Amplification (MLPA) is an application that can be used for the detection of multiple chromosomal aberrations in a single experiment. In one reaction, up to 50 different genomic sequences can be analysed. For a reliable work-flow, tools are needed for administrative support, data management, normalisation, visualisation, reporting and interpretation.

**Results:**

Here, we developed a data management system, *MLPAInter *for MLPA interpretation, that is windows executable and has a stand-alone database for monitoring and interpreting the MLPA data stream that is generated from the experimental setup to analysis, quality control and visualisation. A statistical approach is applied for the normalisation and analysis of large series of MLPA traces, making use of multiple control samples and internal controls.

**Conclusions:**

*MLPAinter *visualises MLPA data in plots with information about sample replicates, normalisation settings, and sample characteristics. This integrated approach helps in the automated handling of large series of MLPA data and guarantees a quick and streamlined dataflow from the beginning of an experiment to an authorised report.

## Background

In medical research, knowledge of chromosomal deletions or amplifications is of great importance. For example, it can help us better understand the genetic causes of certain diseases and as a consequence, improve the treatment and prognosis of individual patients. Classic techniques for the detection of chromosomal abnormalities include karyotyping, Southern blotting, Fluorescent In Situ Hybridisation (FISH), CA-repeat analysis and quantitative micro satellite analysis by real-time PCR [1-4]. In recent years, high-throughput methods based on BAC arrays, SNP arrays and related techniques have gained prominence [5,6]. Although these are excellent tools for whole genome analysis, these techniques are laborious, time-consuming, difficult to implement, expensive and generate large data sets. The management and interpretation of such voluminous data is not a light task. Multiplex Ligation-dependent Probe Amplification (MLPA) [7] has been introduced as a relatively cheap and fast method to perform quantitative chromosomal analysis of up to about 50 genomic DNA or RNA sequences, which is able to distinguish sequences differing in only one nucleotide. This technique fills the gap between the methods that investigate a single locus and the techniques that interrogate thousands of loci.

MLPA is a quick and cost effective approach to testing for the presence of gene deletions or obtaining tumour profiles on multiple loci in a single tube, which can easily be applied in molecular pathology. Furthermore, MLPA only requires small amounts of DNA. Moreover, DNA obtained from formalin fixed paraffin embedded material can be used. Currently, MLPA is used for the validation of array-based comparative genomic hybridisation (array-CGH) and SNP arrays [7-12]. Other applications for MLPA include methylation status determination, copy number analysis in segmentally duplicated regions, expression profiling, and transgene genotyping [13]. The principle of MLPA is that for each locus, two DNA oligonucleotides (probes) must hybridise to their complementary target sequences on the template DNA for ligation to occur. Subsequently a PCR reaction is performed on the ligated probes. After PCR, an aliquot of the PCR product is combined with an internal size marker and deionised formamide. The sample is then injected into a capillary of an automated sequencer, where after a 30 minutes run, the data are subsequently collected for further analysis. Since, the amount of ligated probes is dependent on the number of specific primer binding sites, this method is suitable for the detection of chromosomal deletions or amplifications [7]

The analysis, visualisation and data management of hundreds of samples with many different probes per reaction can be cumbersome. Like in many modern techniques, the results of an MLPA analysis are delivered as lists of values that can be easily imported into spreadsheet applications. Large collections of individual spreadsheets are not the best way to collect and analyse data, especially in an environment where a controlled work flow has to be guaranteed. A database system offers advantages such as the tracking of material used in the tests and consistency in the handling of test results. Some of the information that needs to be managed includes: the origin of normal and test samples, the experimental setup, the identity of the probes, and the quality settings. Normalisation has to be performed within and between samples and results have to be visualised and stored. Sophisticated tools are needed to facilitate the reliable use of MLPA [7,9,14-16]. In this paper, we present a statistical technique for the normalisation of MLPA data and the software component that we have developed to make MLPA a simple, effective, and attractive tool. Here we describe *MLPAinter*, for MLPA interpretation, a system that stores results, instrument settings and sample descriptions in a Microsoft Access database. A special front-end, written in the Borland Delphi language, allows the user to interrogate the database, normalise data and visualise results as heat maps and specialised plots.

## Implementation

MLPA probe kits are obtained from MRC-Holland (Amsterdam, The Netherlands). All assays are performed according to the manufacturers' protocols on an ABI DNA sequencer (Applied Bio Systems, Foster City, CA, USA). *MLPAinter *was constructed using Delphi 2009 (Embarcadero, San Francisco, CA, USA) for the GUI, and Microsoft Office Access 2003 (Microsoft, Seattle, WA, USA) for the standalone database. The runtime requirements for the application are Windows XP or newer. Statistics used in *MLPAinter *as described below, were validated in a series of oligodendroglial tumours as previously described [9]. The source code and a step by step protocol to use *MLPAinter *together with showcase sample files and analysis tables can be obtained from http://code.google.com/p/mlpainter/

## Results

### Data pre-processing

*MLPAinter *can not handle the raw electrophoresis signal and therefore requires that the MLPA amplification product peaks have already been linked to the corresponding MLPA probes of the used MLPA kit. After electrophoresis, all MLPA sample trace files should be pre-processed in standard software for basic analysis of MLPA traces. Subsequently, the report files can be imported. Here we used GeneMapper (Applied Bio Systems, Foster City, CA, USA) for *MLPAinter*, but the system can also import data from the combination Genescan Analysis and Genotyper software (Applied Bio Systems, Foster City, CA, USA). Adaptations to other software programs like Genemarker (Softgenetics, State College, PA, USA) should be straightforward. A step-by-step vignette for Genemapper settings can be found at http://code.google.com/p/mlpainter/. Briefly, the product lengths of the ligated probes are defined with an internal size standard. The peak height and area are calculated for every peak present in the trace. Any undefined peaks are discarded from further analysis. Data tables are then automatically generated with length, height and area of all recognised peaks. These tables are exported from the Genemapper software package and imported into *MLPAinter *for specific analysis of the raw data. Protocols for linking output files from other software packages are planned for future versions.

### Data management

Here, we developed a relational database using Microsoft Access to manage all pertinent information for MLPA experiments and created a front end with Borland Delphi to guide laboratory workflow and data analysis. Characteristics such as the sample number and status, e.g., tumour or normal, DNA concentration and, if available, tumour percentages that are relevant for the performance of the MLPA should be stored in a database. Annotation information like the chromosomal position and gene names of the different probes in a kit should be available for the interpretation of the results in output tables, heat maps, and plots. To assist the laboratory work-flow, electronic and paper sample sheets can be prepared for the automated sequencer. The raw data of the sequencing reports are imported into the database for subsequent quality control steps and analysis.

The relational database contains three hierarchies which are interconnected. The hierarchies are MLPA kits and probes, electrophoresis results, and analyses. In the database tables, next to the specific Kit information, you can find gene and probe names, as well as the physical and cytogenetic location of the probes. All probes in a particular kit are numbered from 1, for the probe with the smallest product size to n, for the probe with the largest product size. Every kit contains a number of probes that can be used for a quality check of the trace. The corresponding products are named based on their size in base pairs. The different kits as defined by MRC-Holland, can be imported from http://www.mlpa.com.

Both the MLPA run and analysis hierarchy use the samples table. This table contains clinical information like the origin of the used DNA, e.g., if the DNA is isolated from whole blood, fresh frozen tissue or formalin fixed paraffin embedded tissue. Every sample is labelled with an N for Normal, T for Test or the Tumour origin of the tissue. Normal samples are treated differently from test samples in the normalisation and analysis steps as described in the normalisation section. An electrophoresis run typically consists of a sample plate to be processed by the sequencer. The sample, the kit, and a unique name for the plate are recorded for each position on the sample plate. Different types of kits can be used within one run. From this information a sample sheet or configuration file is created for the sequencer. The resulting peak heights and peak areas of an MLPA run are imported for all of the probes in a kit and the analysis settings can be set to analyse peak heights or peak areas.

During analysis, specific MLPA runs can be combined from one or more electrophoresis runs. A group of reference probes can be copied from another analysis with the same kit, and can be adapted to suit the needs of the specific analysis. However, to avoid inter experimental differences, values from experiments performed at a different time should not be used. Probes can also be excluded from the analysis. Successful analysis can be finalised by authorising the results. After authorising the analysis, all options are fixed except for visualisation and sorting options.

### Quality control

**Figure 1:**
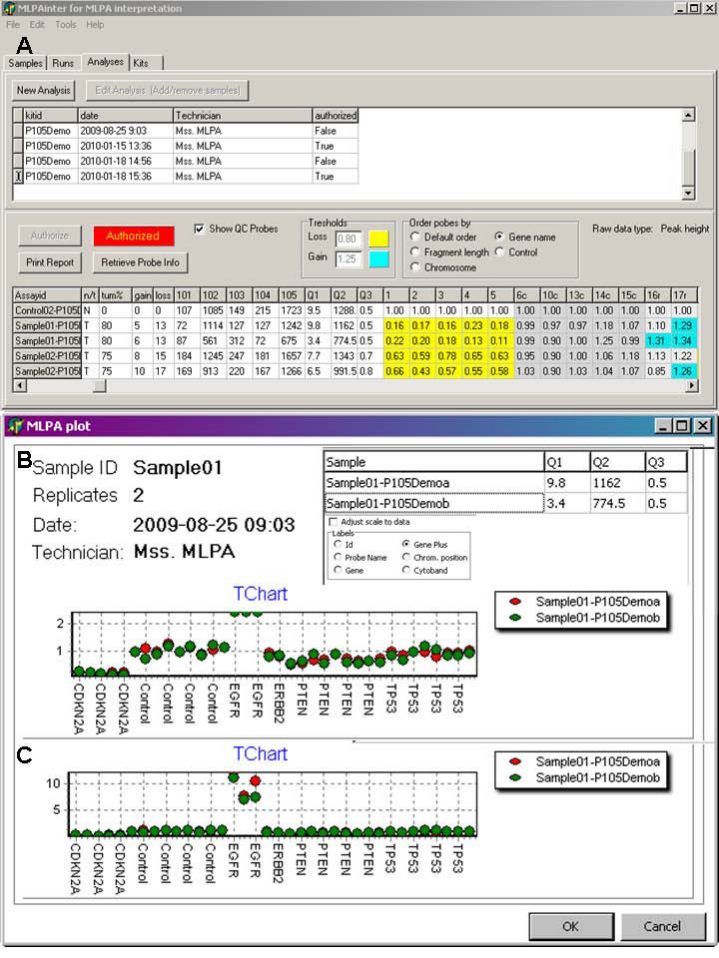
**MLPAinter for MLPA interpretation**. Panel A: Heat map of an authorised series of samples after normalisation. The probes are sorted by the name of the gene. The gain and loss columns show the total number of probes with gain or loss. Q1-3 show the different quality scores calculated from the DNA dependent probes (marked 101-104) and the ligation dependent probe (105) and as explained in the text. Dark grey cells: calibration probes, the id has a suffix 'c'. Light grey cells: reference probes, the id has a suffix 'r'. Yellow cells: probes with loss of one allele (< 0.8). Blue cells: probes with gain of one allele (>1.25). Panel B: Sample plot of an individual sample after normalisation. The quality indices for each replicate are shown. Replicates are visualised in different colours. Probes are sorted by the gene name combined with the chromosomal position. The standard scale can be adjusted in case of samples with amplified probes (see panel C).

MLPAinter presents three data quality indicators, Q1, Q2 and Q3, (Figure 1A) to assist with the decision of whether to include a trace in the analysis.

**Figure 2:**
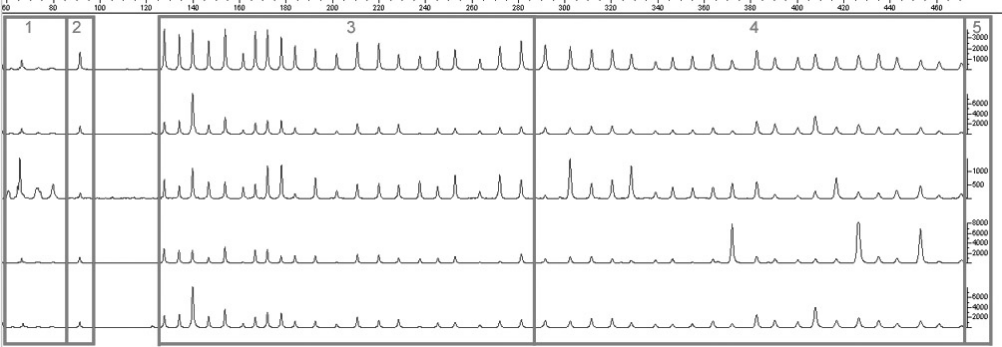
**MLPA sample trace files**. Overview of 5 different sample traces obtained with MLPA kit P105 (Oligodendroglioma-2) showing the necessity of data normalisation. Differences in and between samples are hard to distinguish. Quality aspects of every trace are visible. Probe lengths in base pairs are shown on the x-axis. Box 1: four no template control peaks of 64, 70, 76 and 82 bases, respectively. Box 2: a 94 base pair ligation control peak. Box 3 and 4: larger peaks in the first half than in the second half of the sample trace. Box 5: peak heights are noted on the y-axis

The first indicator (Q1) is the ratio between the ligation dependent peak at 94 base pairs and the median of the DNA dependent 64, 70, 76 and 82 peaks (Figure 2). Van Dijk et al. [10] state that this ratio should be greater than 5 to obtain good and reproducible results. Nonetheless, we have observed that in some cases, lower ratios can also give reliable peak patterns (Figure 2).

The second quality indicator (Q2) is the median peak height of the probe signals present in the kit. If the median of the first 20 ligated probe peak heights is below 450 relative fluorescent units (RFU), the trace quality is considered low. Moreover, because of the limits in the detection optics of the instrument, a median peak height over 4000 RFU is indicative that the trace quality is low (Figure 2) [14].

For the last indicator (Q3) all analysis peaks are split in 2 parts based on sequence length. The value is computed as the median signal of the longest probes divided by the median signal of the shortest probes. Often the longest probes show lower signals, however in high quality traces this indicator is usually over 0.5.

Other factors that are important for the assessment of quality, which can optionally be stored into the database, are the DNA concentration of the sample, the tumour percentage of the tumour specimens and the intrinsic DNA quality of the sample. The combination of these quality parameters allows the user to decide on inclusion or exclusion of a trace from the analysis.

### Normalisation

Raw MLPA results are not calibrated. Peak areas or heights are dependent on sample quality, hybridisation parameters and instrument settings. To analyse the MLPA traces, internal and external control loci are used for the normalisation of the data. External controls, e.g., normal tissue in tumour analysis, have to be present in every experiment for the pattern comparison. Internal controls for the calibration of the samples are present in every kit and are supposed to be non-altered or reference probes in a tumour sample. These reference probes are compared to the probes where DNA changes are expected.

The top trace in Figure 2 shows a normal sample. It is evident that peak heights or areas differ between probes; and these differences have to be corrected. Also the average peak areas or heights may differ from sample to sample. Therefore, sample calibration and probe calibration have to be performed. Consider the data as a matrix *Y*, with columns for the probes and rows for the sample. Then, we need to apply normalisation to both rows and columns. Normalisation is implemented as division by row parameters *r*_*i*_, *i *= 1 ... *m *and column parameters *c*_*j*_, *j *= 1 ... *n*, such that a matrix *X *= [x_jj_] results, with x_jb _= y_jb_/(*r*_*i*_*c*_*j*_). We prefer to work on the original scale instead of with logarithms because loss and gain correspond to integer ratios (including zero) on the original scale.

A simple approach would be to take row and column medians for *r *and c, *respectively*. This could work well if the number of deletions or amplifications is relatively small. However, for samples with a large number of deletions (more than 50%), the corresponding row median might become a number near zero and normalisation by dividing with this small number would give a completely wrong result.

To improve normalisation and obtain calibration factors, we use only a subset of the samples and probes. Specifically, we use normal samples, and only a subset of the probes, where copy number changes are unlikely, even in tumour samples. We use the following algorithm:

1. To correct for the sample-to-sample variation, divide the peak heights or areas of all the probes in each sample by their median. This gives provisional row parameters, *ř *for the normal samples, and provisional normalisation of the normal samples.

2. To correct for systematic differences between probes, divide the peak heights or areas of all the probes within a MLPA run by their median. This results in the *normalised peak areas or heights*, and represents the column parameters *c *for all probes. The average of all probes is now close to 1.

3. Select the probes that have a small probability of change in copy number. Call these *the reference probes*. The remaining probes are called the *focus probes*, since we look for changes in these. The description file for commercial kits includes this information, and the program uses these probes by default.

4. Select the part of the data that represents the normal control or non-tumour samples and the reference probes.

5. Redo steps 1 and 2 for the subsets of reference samples and reference probes.

6. Determine which probes are most stable. Subtract 1 from each normalised peak height or area and take the absolute value. Compute for integrated MLPA analysis the median of these numbers for each probe. This is the median of the absolute deviations: MAD.

7. The reference probes with the lowest MAD are most stable. Select the five probes closest to zero. These are the probes that we call the *calibration probes*.

8. Compute the median peak height or area of the 5 calibration probes for each sample (normal and test samples or tumours and non-tumours). Divide all peak heights or areas as computed in step 2 in each sample by this value. This gives the final row parameters *r *for all samples, and their final normalisation.

### Reference probe selection

As in quantitative RT-PCR, the selection of reference probes is a critical element of the analysis [17]. MLPA kits contain about 10 reference probes that are includ for normalisation purposes because they are not involved in the experimental hypothesis/diagnostic question. Alternatively, one can usually find a subset of probes in existing kits that are known not to be involved in the hypothesis. The procedure selects the most stable probes from the reference probes to calibrate the data. The number of calibration probes used (five, in this instance) did not significantly influence the results (data not shown). However, the number is configurable in the program. If probes show high variability between replicates or between normal samples, they should be excluded from the analysis.

### Visualisation

We have designed a number of visualisations to interpret the results after the normalisation and quality control of the data set. The first visualisation is a heat map that shows all of the data in an experiment. Deletions and gains are colour-coded with configurable thresholds. Probes can be sorted by locus names or chromosomal position. The reference and calibration probes are clearly differentiated by a grey-shade (Figure 1). Another visualisation shows the normalised values of all replicates of one sample in a plot (Figure 1). Technical replicates are shown in different colours. On the x-axis, the different probes are shown in the selected probe order. The y-axis is on a scale from 0 to 2.5, where 0 stands for absent probes. Ideally probes at genomic loci with loss of a single allele show values around 0.5. Unaltered probes are visualised around 1.0. Probes with DNA gains have values around 1.5 or above. In tumour samples contaminated with normal DNA these values are usually not that outspoken. The researcher should keep this in mind during the interpretation. Information about sample characteristics and probes used are also shown in the plots.

### Future developments

Currently the system is suited for the analysis, visualisation and data management of MLPA. However, all of the information generated during an experiment is still not fully integrated in the data analysis. For instance, tumour percentages can be stored in the database and will be displayed, but it is up to the user to incorporate this information into the interpretation. We plan to include the tumour percentage and probably the DNA index for automated identification of the allelic state of the chromosomal aberrations in the analysed sample [18]. Another worthwhile improvement would be to remove the dependency on an external program to do the peak detection.

## Discussion

MLPA has a variety of applications for the detection of changes in dosage at a single locus, e.g., a subtelomeric locus, to those with multiple changes. Up to 50 different probes (genomic sequences) can be interrogated in one single reaction. Advantages are that only small amounts of DNA are needed and that DNA isolated from formalin fixed paraffin embedded material can be used. As it stands, currently available software tools for MLPA analysis do not integrate data management, normalisation and visualisation, and do not always perform adequate data normalisation between and within traces. In these packages, the quality aspects of the analysis are not always taken into consideration. Therefore, we have developed a method for MLPA data interpreting, *MLPAinter*, in which sample information can be stored, and where the laboratory and analysis workflow is assisted. Experiments are prepared by selecting samples and MLPA kits. Then sample sheets for automated sequencers are generated, which can easily be imported in the sequencer, avoiding manual input and typing errors. Analysis tables can then be imported from standard DNA analysis programs. Given the sensitivity and reproducibility of this methodology, the requirements for proper internal controls for normalisation have to be stringent [17]. For that reason, in each kit, the manufacturer has provided sets of reference probes for sample data. However, for the analysis of unpredictable (tumour) samples, these provided reference probes may be inadequate. Thus, we created an algorithm that will select the 5 most stable reference probes and suggest that these probes be used for the normalisation of the traces. The user has full control over the settings of the analysis, and changes like including or excluding samples or designating probes as reference probes result in immediate recalculations. All calculations can be visualised in plots. By authorising the results, the analysis settings are definitively linked to the analysis and can no longer be changed.

MLPA results are, in general, very reproducible. Still, we perform all tests at least in duplicate, especially in a diagnostic setting. *MLPAinter *supports the handling of replicates in the analysis. We previously validated the statistics used for *MLPAinter *on a series of DNAs that were obtained from formalin fixed and paraffin embedded oligodendroglial tumours by correlating the results with those obtained by fluorescent in situ hybridisation (FISH). The MLPA results were reproducible in all samples in which repeated experiments were performed [9].

## Conclusions

We have combined the analysis, visualisation and data management for MLPA in a tool, *MLPAinter *for MLPA interpretation, which makes use of a relational database with a Delphi front end. This integrated approach helps in the automated handling of large series of MLPA data and helps to guarantee a quick and streamlined dataflow from the initiation of an experiment to the generation of authorised report. *MLPAinter *has been successfully used in our lab for over two years to manage over 3000 samples. Moreover, different MLPA kits have been successfully used for this type of analysis, e.g., Kit P088 and P105 for the analysis of Oligodendrogliomas, P024 for CDKN2A/B and P036 for subtelomeric regions.

## Availiability and Requirements

Project name: MLPAinter

Project home page: http://code.google.com/p/mlpainter/

Operating system(s): Windows XP or higher

Programming language: Delphi

Other requirements: no

Licence: GNU GPL 3.0

Any restrictions to use by non-academics: no

## Abbreviations

MLPA: Multiplex Legation-Dependent Probe Amplification; RFU: relative fluorescent units; RT-PCR: Real Time-PCR; MAD: median of the absolute deviations.

## Authors' contributions

RvE specified MLPAinter functionalities and drafted the main parts of this manuscript. PHCE developed the statistics for normalisation of the MLPA data. RN validated statistics on a series of oligodendrogliomas. AMCJ, TvW and JM participated in the study design and supervised the implementation of MLPAinter in molecular diagnostics. JO developed the software and supervised the drafting of the manuscript. All authors read and approved the final manuscript.
